# Differentiation in an inclusive trauma system: allocation of lower extremity fractures

**DOI:** 10.1186/s13017-018-0178-1

**Published:** 2018-04-13

**Authors:** F. S. Würdemann, D. P. J. Smeeing, S. Ferree, F. Nawijn, E. J. M. M. Verleisdonk, L. P. H. Leenen, R. M. Houwert, F. Hietbrink

**Affiliations:** 1 Department of Surgery, Diakonessenhuis Utrecht, Utrecht, The Netherlands; 2 Traumacenter, University Medical Center Utrecht, Utrecht, The Netherlands; 3 Department of Surgery, University Medical Center Utrecht, Heidelberglaan 100, 3584 CX Utrecht, The Netherlands

**Keywords:** Inclusive trauma system, Lower extremity fractures, Level 1, Level 2, Trauma centre, Resource demand, Maturation, Centralization, Triage

## Abstract

**Background:**

Implementation of an inclusive trauma system leads to reduced mortality rates, specifically in polytrauma patients. Field triage is essential in this mortality reduction. Triage systems are developed to identify patients with life-threatening injuries, and trauma mechanisms are important for triaging. Although complex extremity fractures are mostly non-lethal, these injuries are frequently the result of a high-energy trauma mechanism. The aim of this study is to compare injury and patient characteristics, as well as resource demands, of lower extremity fractures between a level (L)1 and level (L)2 trauma centre in a mature inclusive trauma system.

**Methods:**

This is a retrospective cohort study. Patients with below-the-knee joint fractures diagnosed in a L1 or L2 trauma centre between July 2013 and June 2015 were included. Main outcome parameters were patient demographics, trauma mechanism, fracture pattern, and resource demands.

**Results:**

One thousand two hundred sixty-seven patients with 1517 lower extremity fractures were included. Most patients were treated in the L2 centre (L1 = 417; L2 = 859). Complex fractures were more frequently triaged to the L1 centre. Patients in the L1 centre had more concomitant injuries to other body regions and ipsi- or contralateral lower extremity. Patients in the L1 centre were more resource demanding: more surgeries (> 1 surgery; 24.9% L1 vs 1.4% L2), higher immediate admission rates (70.1% L1 vs 37.6% L2), and longer length of stay (mean 13.4 days L1 vs 3.1 days L2).

**Conclusion:**

The majority of patients were treated in the L2 trauma centre, whereas complex lower extremity injuries were mostly treated in the L1 centre, which placed higher demand on resources and labour per patient. This change in allocation is the next step in centralization of low-volume high complex care and high-volume low complex care.

## Background

Lower extremity injuries have a large impact on the functional outcome after trauma and are frequently seen in both high-energy trauma and polytrauma patients, where the impact of these fractures is even more evident [1–4]. Even more, complex injuries, defined by the severity of the sustained injuries or multiple fractures in the ipsilateral extremity, are important factors that determine long-term functional outcome [2, 5]. Complex extremity injuries might require a different approach compared to non-complex injuries. To optimize outcome in complex lower extremity fractures, 24/7 availability of emergency operating teams, timing of delayed reconstructions, and a multidisciplinary approach are deemed essential. On the other hand, fast tracks, elective procedures, and designated physicians might be factors for patient satisfaction in non-complex cases. Therefore, optimizing the distribution of (lower) extremity fractures amongst trauma centres may be beneficial for both patients and trauma systems as a whole.

With improvements in trauma care and the implementation of inclusive trauma systems, mortality rates have declined over the past decades [6, 7]. Allocating patients to the hospital with the most appropriate level of care for their injuries is essential to ensure the best possible outcome and reduce mortality. This applies mostly to patients suffering severe injuries (i.e. polytrauma patients), which is frequently due to high-energy trauma mechanisms. Therefore, trauma mechanism is considered an important factor in field triage systems, in addition to key elements such as the number and severity of sustained injuries and vital signs. Allocation of polytrauma patients to a level 1 (L1) trauma centre is beneficial because of specialized care and in-hospital trauma pathways, as a result of the anticipated high demand on resources and labour intensity [6]. Conversely, less complex injuries tend to require non-urgent, or less urgent interventions, resulting in more efficient use of available resources. Therefore, these injuries are more suitable for treatment in a high-volume level 2–3 (L2) trauma centre.

The allocation of lower extremity fractures within a trauma system has not been described previously. However, volume-outcome relationships have been well established in recent literature [8–11]. Both super-specialisation and logistical arguments might guide differentiation of low complex cases to high-volume centres whilst high complex cases differentiate to specific care facilities such as L1 trauma centres. This is especially the case in a densely populated area with multiple hospitals within a short distance apart [9]. In a mature inclusive trauma system, patients with complex extremity fractures may be preferably allocated to a L1 trauma centre, while a larger number of patients overall may be allocated to a L2 centre. The aim of this study is to compare patients and resource demands of lower extremity fractures between a L1 and L2 trauma centre in a mature inclusive trauma system.

## Methods

### Setting

A multicentre retrospective cohort study was performed in a level 1 trauma centre hospital (L1) (University Medical Center Utrecht) and a level 2 trauma centre hospital (L2) (Diakonessenhuis Utrecht); both situated in the central region of the Netherlands. Trauma care in the Netherlands is divided into 11 trauma regions. In 2015, the central region covered more than 1.5 million inhabitants and consisted of one L1 trauma centre; the University Medical Center Utrecht (UMCU), plus five L2 hospitals and 12 outpatient clinics, all categorized according to the available resources. This is a mature inclusive trauma system as documented previously [8, 9, 11–13]. The distance between these L1 and L2 centres is 5 km maximum or a 10-min ambulance drive. The L1 has 8 trauma surgeons with 24/7 in-house coverage for initial resuscitation, surgery (general and orthopaedic), and intensive care consultancy. The L2 centre has 3 trauma surgeons on call during shift hours. At the L2 centre, initial resuscitation is delivered by an emergency physician during office hours and by a general surgery house officer during shift hours (5 PM—8 AM during weekdays, plus 24 h on weekends).

### Field triage

The ambulance personnel in this region use the nationwide Dutch emergency service protocol to decide the designated level of trauma care [14]. Indications to transport trauma patients to a 1 trauma centre are compromised airway, breathing or circulation; a GCS < 9 or decreasing, anisocoric pupils; neurological deficits (to more than one extremity); and hypothermia (≤ 32 °C). Injury-specific criteria are a penetrating injury to the head, thorax, or abdomen, flail chest, pelvic instability, ≥ 2 fractures (to femur, tibia, and humerus), and amputations proximal to the ankle or wrist. In addition, paramedics may triage a patient after high-energy trauma directly to the L1 trauma centre without immediate identification of any of the aforementioned injuries [15].

### Patients

The focus of the current study was on patients with a below-the-knee fracture. All patients, both patients with an isolated fracture below the knee and polytrauma patients, diagnosed with at least one fracture below the knee between July 2013 and June 2015 were included. Inclusion criteria were age ≥ 18 years and initial presentation or referral to the L1 or L2 centre. Exclusion criteria were age < 18 years; ligamentous injuries, including avulsion fractures, Jones fractures, and tibial eminence fractures; isolated phalangeal fractures; pathological or stress fractures; incomplete radiographic imaging; and treatment received elsewhere. Patients who were transferred to or treated in another hospital were regarded as lost to follow-up, but their fracture classification and trauma mechanism were collected. Patients transferred between our L1 and L2 centres due to a capacity issue or patient preference were scored based on the hospital of initial presentation. Patients transferred between our L1 and L2 centres for a medical indication were scored based on the hospital where definitive treatment was performed.

### Data extraction

Data were derived from the electronic patient documentation (EPD) and the Dutch National Trauma Database (DNTD) of both hospitals. The DNTD is a prospectively collected database including data on all admitted trauma patients. Prehospital transport, immediate transport to the operation room (OR) or intensive care unit (ICU), and Injury Severity Score (ISS) were collected from the DNTD. Patient characteristics such as gender, age at time of trauma, fracture details, and length of hospital stay in days were collected from the EPD [16, 17].

Trauma mechanisms were categorized as fall from height < 3 or ≥ 3 m, sport-related injury, crush injury, suicide attempt, traffic accident (divided into car, motor, scooter, bicycle, pedestrian), or other. When an isolated trauma mechanism was specified, it was categorized as such: simple sprain, direct impact, or penetrating injury. Trauma mechanism was classified as low-energy trauma (LET) or high-energy trauma (HET) according to the criteria derived from the ATLS guidelines, see Table 1 [18]. Many definitions of polytrauma are used [19]. In this study, polytrauma was defined as an ISS > 15. For patients who did not need clinical admission, an ISS < 15 was noted. The energy of the trauma was classified as ‘unknown’ in case of insufficient information.

**Table 1:** Criteria for HET

Fall from height ≥ 3 m or ≥ 3× body length	
Car accident - > 65 km/h - Vehicle intrusion passenger compartment > 30 cm - Vehicle rollover - Passenger ejection from vehicle - Fatality in same vehicle	
Motor or scooter accident > 32 km/h	
Car-pedestrian or car-bicycle impact > 8 km/h	
Suicide attempt (any)	
Crush injury	
Direct impact by blunt object either heavy or at high velocity	
Penetrating objects (high velocity)	

Non-operative and operative treatment was documented for each patient and each fracture. Interventions were categorized as primary, secondary, or complication-related surgery. Open reduction internal fixation, closed reduction internal fixation, and external fixation were considered primary surgeries. Replacing external fixation with internal osteosynthesis, revision or removal of internal osteosynthesis within a year, and flap or split skin surgery for soft tissue damage were categorized as secondary surgeries. Debridement in case of an infection, including vacuum dressing placement or replacement in the operating room (OR), correction of angulation, arthrolysis, fasciotomies, and neurolysis were deemed complication-related surgeries.

To define fracture patterns, the lower leg was divided into eight regions using the rule of squares [20]. The tibial shaft, distal tibia, calcaneus, talus, midfoot, and metatarsals were classified by a simplified OTA-AO (Orthopedic Trauma Association-Arbeitsgemeinschaft für Osteosynthesefragen) system [20]. Ankle fractures were classified using the uni-, bi-, and tri-malleolar Weber classifications, and tibial plateau fractures using the Schatzker classifications [21–23]. Three researchers (FW, DS, and SF) reviewed all radiographic studies and classified the fractures. In case of disagreement, the fractures were classified by two consultant (orthopaedic) trauma surgeons (MH and FH).

The level of soft tissue damage in open fractures was classified using the Gustilo classification system [24, 25]. An extremity was considered mangled when a fracture and 2 of the following criteria were present: nerve, vascular, and/or severe soft tissue damage [20, 26]. Nerve or vascular damage information was retrieved from the physical examination reports in the EPD.

### Data analysis

Statistical analysis was performed with SPSS version 21 for Windows. Parametric tests were used when > 30 items per group were compared. In all other cases, non-parametric tests were used. The *χ*^2^ test was used for categorical variables, and Fisher’s exact test was used for categorical variables with a cell volume less than 5. Results were presented according to exact count and percentages of total patients or fractures within a specific trauma centre. Non-parametric variables were analysed using the Mann-Whitney test and shown as median and range. The Kruskal-Wallis test was used when two or more groups were combined with a continuous scale. A *p* value of ≤ 0.05 was considered significant.

## Results

### Demographics

**Fig. 1:**
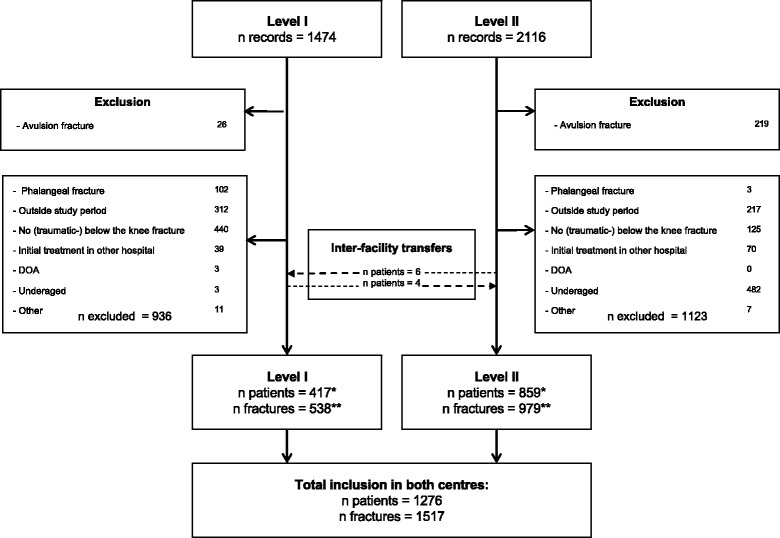
Inclusion and exclusion scheme. *Patients lost to follow-up: L1 *n* = 23, L2 *n* = 56. **Fractures lost to follow-up: L1 *n* = 29, L2 *n* = 65

In this study, 859 patients with a below-the-knee fracture were included in the L2 centre and 417 in the L1 centre (Fig. 1). One thousand five hundred seventeen fractures were analysed: 979 in the L2 centre and 538 in the L1 centre. Two hundred nineteen patients were excluded in the L2 centre and 26 patients in the L1 centre due to avulsion fractures.

**Fig. 2:**
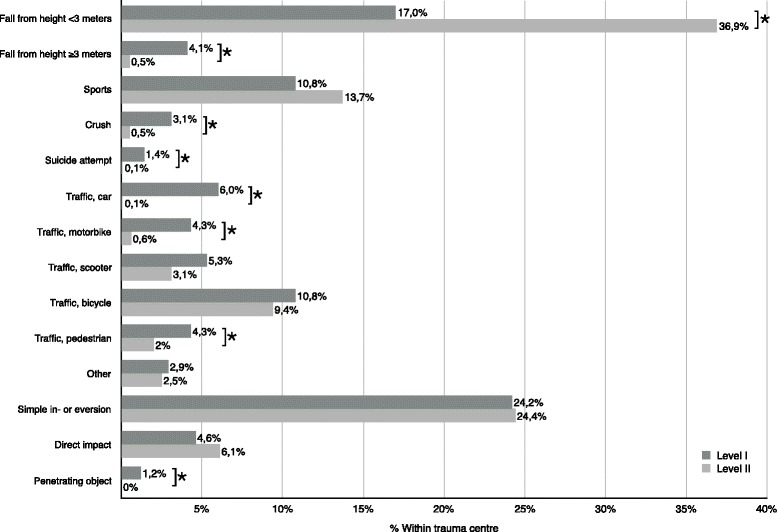
Trauma mechanism frequencies per level trauma centre. Significant difference: *p* value of < 0.005

Eight patients died during admission, all of which were in the L1 centre. Three died due to their injuries (2 intracranial haemorrhage, 1 hypovolemic shock) and 5 for other reasons (myocardial infarction, cerebral vascular accident, gut ischemia, 2 unknown). Six patients were transferred between the two facilities: 4 patients from the L2 to the L1 and 2 from the L1 to the L2. Patient characteristics are presented in Table 2. In the L1 centre, 32.6% of the patients had injuries due to a high-energy trauma (HET) compared to 3.5% in the L2 centre (*p* < 0.001) (Fig. 2). Of all fractures, 205 patients (218 fractures) were operated on in the L2 centre and 202 patients (259 fractures) in the L1 centre.

**Table 2:** Patient characteristics per level trauma centre

	Level I		Level II		*p* value
	*n* patients = 417		*n* patients = 859		
Male gender (*n*, %)	205	(49.2%)	347	(40.4%)	0.005
Age (mean, SD)	47	(19)	51	(20)	< 0.001
Multiple fractures (*n*, %)	67	(16.2%)	70	(8.2%)	< 0.001
Bilateral fractures (*n*, %)	24	(5.8%)	5	(0.6%)	< 0.001
Fractures per patient (mean, SD)	1.3	(0.8)	1.1	(0.5)	< 0.001
ISS > 15 (*n*, %)	55	(13.3%)	1	(0.1%)	< 0.001
HET mechanism (*n*, %)	136	(32.6%)	30	(3.5%)	< 0.001
Out-of-hospital on site medical care delivered by MMT (*n*, %)	17	(4.1%)	0	(0%)	< 0.001
Emergency admission on day of trauma (*n*,% of admittances)	169	(70.1%)	88	(37.6%)	< 0.001
Patients requiring admission for treatment (*n*, %)^1^	241	(61.2%)	234	(29.2%)	< 0.001
Direct transfer to OR and ICU (*n*, %)	33	(7.9%)	0	(0%)	< 0.001
Length of first hospital stay in days (mean, SD)	13.4	(18.9)	3.1	(4.5)	< 0.001
First hospital stay > 1 week (*n*, % of admittances)	126	(52.3%)	40	(17.1%)	< 0.001
Patients surgically treated (*n*, %)	202	(51.3%)	205	(25.6%)	< 0.001
Patients treated with > 1 surgery (*n*, %)	98	(24.9%)	11	(1.4%)	< 0.001
Surgeries on lower extremities per patient (mean, SD)	1.1	(1.7)	0.3	(0.5)	< 0.001

*HET* high-energy trauma, *MMT* mobile medical team (helicopter)

^1^Both direct admissions and admissions for postponed surgeries

### Fracture demographics

**Fig. 3:**
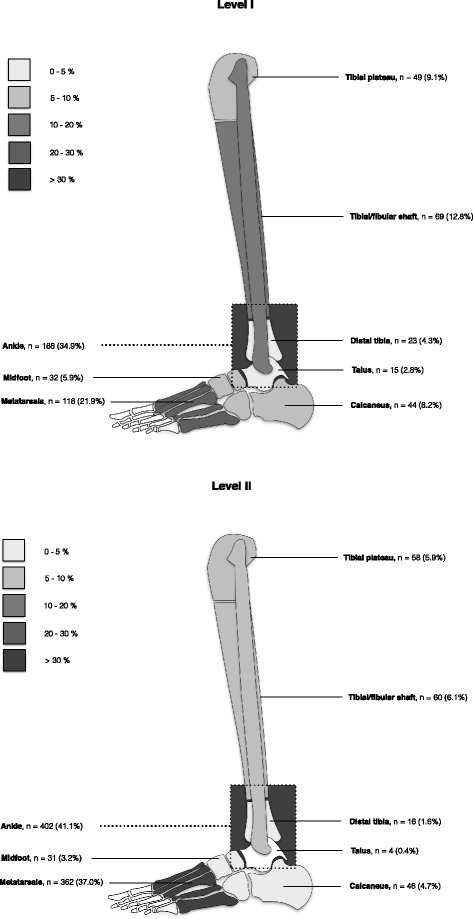
Fracture frequencies per anatomical region (level 1 and level 2)

Ankle and metatarsal fractures were the most prevalent fractures in both centres (Fig. 3), accounting for 70.5% of all fractures (590 ankle fractures and 480 metatarsal fractures total). Most of these were seen in the L2 centre (metatarsal fracture *n* = 362 and ankle fracture *n* = 402). The most infrequent fractures were talus fractures (1.3%) and distal tibia fractures (2.6%). Based on the registry, 219 patients in the L2 and 26 patients in the L1 suffered minor or non-fracture injuries (e.g. avulsion fractures).

### Fracture classification

A difference in fracture classification between the L1 and L2 centres was observed for tibial plateau, tibial shaft, ankle, and foot fractures. The presentation of more complex tibial plateau fractures (Schatzker types 4–6), complex tibial shaft fractures, distal tibia fractures, displaced calcaneal fractures, talus fractures, and complex midfoot fractures (Chopart, Lisfranc, and navicular/nutcracker) was significantly greater in the L1 centre.

Ankle fractures were seen more frequently in the L2 centre and accounted for almost half of the total amount of fractures treated (*n* = 402, 41.1%), of which the majority were uni-malleolar fractures (72.4% within L2). Metatarsal fractures were also more often treated in the L2 centre (*n* = 362, 37.0%). A complete overview of the fracture distribution is shown in Table 3.

**Table 3:**
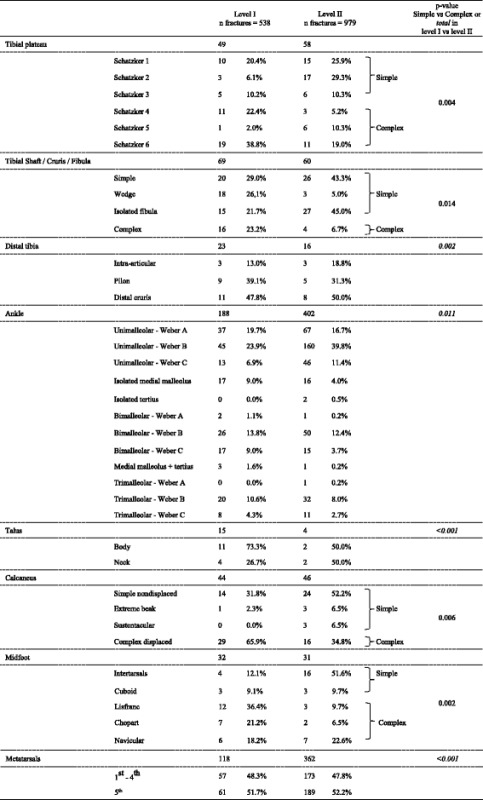
Fracture classifications and complexity per level trauma centre

Fractures were more often associated with concomitant injuries in the L1 centre (*p* < 0.001) (e.g. soft tissue, vascular, and neurological damage). The L1 centre treated more open fractures (*n* = 72 or 13.4% versus *n* = 15 or 1.5%, *p* < 0.001) and more mangled extremities (*n* = 15 or 3% versus *n* = 0 or 0%, *p* < 0.001) than the L2 centre. Fracture characteristics are shown in Table 4.

**Table 4:** Fracture characteristics and treatment per level trauma centre

		Level I		Level II		*p* value
		*n* fractures = 538		*n* fractures = 979		
Fractures surgically treated (*n*, %)*		259	(50.9%)	218	(23.9%)	< 0.001
ORIF/CRIF (*n*, %)*		216	(42.4%)	216	(23.7%)	< 0.001
Fractures treated with external fixation (*n*, %)*		43	(8.4%)	2	(0.2%)	< 0.001
Fractures requiring secondary surgeries (*n*, %)*		64	(12.6%)	9	(1.0%)	< 0.001
Fractures requiring complication surgeries (*n*, %)*		55	(10.8%)	6	(0.7%)	< 0.001
Open fractures (*n*, %)		72	(13.4%)	15	(1.5%)	< 0.001
Gustilo classification (*n*, %)	1	10	(1.9%)	8	(0.8%)	
	2	18	(3.3%)	2	(0.2%)	
	3a	17	(3.2%)	4	(0.4%)	
	3b	19	(3.5%)	1	(0.1%)	
	3c	8	(1.5%)	0	(0.0%)	
Mangled extremity (*n*, %)		15	(2.7%)	0	(0.0%)	< 0.001
Nerve damage (*n*, %)		11	(2.0%)	3	(0.3%)	< 0.001
Vascular damage (*n*, %)		15	(2.7%)	1	(0.1%)	< 0.001
Fasciotomy (*n*, %)*		13	(2.5%)	0	(0.0%)	< 0.001
Vascular repair needed (*n*, %)*		5	(1.0%)	0	(0.0%)	0.006
Soft tissue coverage (*n*, %)*		16	(3.1%)	0	(0.0%)	< 0.001
Amputations*		11		0	(0.0%)	< 0.001

*ORIF* open reduction internal fixation, *CRIF* closed reduction internal fixation

*Percentages are based on level I *n* = 509 and level II *n* = 914, excluding fractures lost to follow-up

### Resource utilization

Most fractures were seen in the L2 centre, and these were generally treated with cast immobilization. In total, 50.9% of the fractures in the L1 centre were treated surgically compared to 23.9% in the L2. The total number of patients undergoing surgical fixation was comparable for the L1 and L2 (*n* = 216 and *n* = 216). The incidence of secondary surgeries and complication-related surgeries was higher in the L1 centre (*p* < 0.001 and *p* < 0.001). All patients that required soft tissue coverage or primary vascular repair were treated at the L1 centre. More patients were immediately admitted to the L1 centre (70.1% on the day of trauma compared to 37.6% in the L2). The hospital length of stay (HLOS) in the L1 was significantly longer than in the L2 centre, and 52.3% of the patients in the L1 had a HLOS > 1 week (Table 2).

## Discussion

In this mature inclusive trauma system situated in a densely populated area, most fractures were treated in the L2 centre. The majority of fractures overall (including avulsion fractures) were treated in the L2 centre; the amount of surgical procedures performed per surgeon was higher in the L2 centre, run by a smaller surgical team. The majority of complex fractures were treated in the L1 centre. This was demonstrated by the number of immediate surgical procedures, late secondary surgeries, a necessary multidisciplinary approach, and utilization of additional resources. Fracture classification supported this difference in complexity, as did accompanying soft tissue injury and concomitant injuries to the lower extremity and other body regions.

The presented differentiation in this inclusive trauma system occurred spontaneously, without regional contracts or prehospital guidelines. Several factors may have contributed to this process. First, the prehospital triage guidelines are aimed at the identification of polytrauma patients with potentially lethal injuries and not so much at (isolated) complex lower extremity injuries. These guidelines in combination with some options for the paramedics that allow direct triage to the L1 centre resulted in the allocation of 78% of the polytrauma patients to the L1 centre in our region as demonstrated previously [15]. Trauma mechanism is one of these options in the triage decision making by paramedics in our country, which may have contributed to the described differentiation in allocation of lower extremity fractures. Second, an important factor that may have contributed to this differentiation is the relatively short distance between the L1 and L2 centres, which is applicable not only to the Central Region of the Netherlands, but also to other densely populated areas with multiple established hospitals. This is the case in most large metropolis cities around the world. The geographic localisation might also have contributed to the difference, as the L2 centre is within the centre of the city, while the L1 centre is situated on the edge of the urban area. Last, in a thrive to improve patient care, the L1 and L2 centres optimized their profile and logistics in accordance to their designated role in the inclusive trauma system. The L2 centres implemented fast tracks for specific diagnoses (e.g. ankle fractures/distortion, wrist injuries, hip fractures), with one surgeon responsible for the patient’s whole course including clinical care, surgery, post-operative consultation, and outpatient follow-up. In contrast, the L1 centre implemented 24/7 specialist care, emergency operating team availability within minutes, and flexible OR time on a daily basis.

Differentiation and specialisation leads to optimization of care. Although there is a diversity in injury type with regard to the complex injuries, the required treatment regime, necessary resources, and flexible logistics make acute complex care of lower extremity fractures a separate entity that requires its own differentiation. Complex lower extremity fracture types were more frequently treated in the L1 centre. Tibial plateau Schatzker types 4 through 6, complex tibial shaft, displaced calcaneal, and complex midfoot fractures were more prevalent in the L1 compared to the L2 centre (Table 3). It is tempting to speculate that these fractures are more frequently the result of high-energy mechanisms and therefore predisposed to presentation to an L1 centre. This is supported by trauma mechanism analysis, as all high incidence complex fracture types in the L1 were related to HET mechanisms, such as a fall from height (e.g. suicide attempts), traffic, and crush mechanisms [27]. This is further illustrated by a higher incidence of open fractures, an increased need for external fixations, fasciotomies, amputations, and other secondary or complication surgeries in the L1 centre. Open lower extremity fractures have a greater likelihood of infection, which makes early soft tissue coverage within hours to days desirable. Vascular damage and neurological damage ought to be treated urgently [28, 29]. Furthermore, soft tissue damage especially in the case of multiple non-contiguous fractures often requires serial operations [30]. As a result, there is a high demand on multiple resources in the L1 centre. In total, 24.9% of all fractures in the L1 required more than one operation. In contrast, only 1.4% of the patients in the L2 centre underwent multiple procedures. The presence of an in-hospital trauma surgeon as well as a dedicated OR team for complex and severely traumatized patients was initiated to improve outcomes, but adds to the costs incurred per patient [31–33]. Although not all secondary and complication-related surgeries were performed during the initial hospitalization, also in a semi-acute setting, they put pressure on OR capacity and require personnel with a high level of expertise. OR utilization is one of the costliest components in secondary care [34]. As a consequence, treatment of the complex extremity injuries is expensive.

Additionally, the high incidence of immediate admissions on the day of trauma and longer lengths of hospital stay reveal a striking difference between the L1 and L2 centre. The HLOS of patients admitted to the L1 was longer than 1 week in more than 50% of patients. In total, 271 patients (65%) were presented with an isolated injury below the knee in the L1 centre. However, as 50% of the patients required admission over a week, additional injuries (146/417 = 35%) cannot solely explain the difference in HLOS. More likely, the complexity of the injuries explains the difference in admission rate, HLOS, and resources used in the L1 centre. Almost the same number of patients required admission to the L2 centre as the L1 centre, resulting in a high turnover rate in the L2 hospital. Optimization and maturation of this inclusive trauma system is important not only in reducing morbidity and mortality, but also in optimizing management of resources.

Recent studies on fracture epidemiology show an increasing incidence of extremity fractures with a trend towards surgical treatment, possibly due to development in surgical techniques [35–37]. This means a higher demand on resources is to be expected in the near future. A balanced distribution of trauma patients within the inclusive trauma system is of utmost importance in order to optimize logistics, contain costs, and ensure the best clinical outcomes. For specific injuries, official trauma region agreements between centres might prove beneficial, but as this study demonstrated, might not be essential [10].

Limitations of this study are the absence of prehospital triage data which forced assumptions based on ISS scores and trauma mechanisms. Also, surgeon-based considerations in injury management may have influenced type and extent of treatment and are not traceable. Due to the retrospective nature of this study, no data on outcome and fracture union are available. This would have provided additional insight into the outcomes of fractures treated in both trauma centres. Future studies should focus on these subjects.

This is the first study to assess fracture pattern differences in L1 and L2 hospitals in an inclusive trauma system. It provides detailed information on a large number of patients with extensive information on fracture classifications and resource demands. Of great importance is the trauma mechanism, which may have implications on pre- and in-hospital triage. Future studies should investigate factors that influence triage to L1 or L2 trauma centres.

## Conclusion

Most patients were treated at the L2 centre, while complex lower extremity injuries were mostly treated at the L1 trauma centre. Treatment of below-the-knee fractures differed, within the mature inclusive trauma system, between the high-volume low complexity L2 trauma centre and low-volume high complexity L1 trauma centre. Patients with complex injuries placed a high demand on available resources. Trauma mechanism may prove important for further differentiation and optimization of resource utilization.

## Abbreviations

ATLS: Advance Trauma Life Support
DNTD: Dutch National Trauma Database
EPD: Electronical patient documentation
GCS: Glasgow Coma Scale
HET: High-energy trauma
ICU: Intensive care unit
ISS: Injury Severity Score
L1 or L2: Level 1 or level 2 trauma centre
LET: Low-energy trauma
OR: Operating room
OTA-AO: Orthopaedic Trauma Association-Arbeitsgemeinschaft für Osteosynthesefragen
UMCU: University Medical Center Utrecht
